# Spectral Image Processing for Museum Lighting Using CIE LED Illuminants

**DOI:** 10.3390/s19245400

**Published:** 2019-12-07

**Authors:** Miguel Ángel Martínez-Domingo, Manuel Melgosa, Katsunori Okajima, Víctor Jesús Medina, Francisco José Collado-Montero

**Affiliations:** 1 Department of Optics, University of Granada, 18071 Granada, Spain; mmelgosa@ugr.es; 2 Faculty of Environment and Information Sciences, Yokohama National University, Kanagawa 240-8501, Japan; okajima@ynu.ac.jp; 3 Department of Painting, University of Granada, 18071 Granada, Spain; vmedina@ugr.es (V.J.M.); fcollado@ugr.es (F.J.C.-M.)

**Keywords:** spectral imaging, colorimetry, hyperspectral line scanner, CIE illuminants, CIEDE2000

## Abstract

This work presents a spectral color-imaging procedure for the detailed colorimetric study of real artworks under arbitrary illuminants. The results demonstrate this approach to be a powerful tool for art and heritage professionals when deciding which illumination to use in museums, or which conservation or restoration techniques best maintain the color appearance of the original piece under any illuminant. Spectral imaging technology overcomes the limitations of common area-based point-measurement devices such as spectrophotometers, allowing a local study either pixelwise or by selected areas. To our knowledge, this is the first study available that uses the proposed CIE (Commission Internationale de l’Éclairage) light-emitting diode (LED) illuminants in the context of art and heritage science, comparing them with the three main CIE illuminants A, D50, and D65. For this, the corresponding colors under D65 have been calculated using a chromatic adaptation transform analogous to the one in CIECAM02. For the sample studied, the CIE LED illuminants with the lowest average CIEDE2000 color differences from the standard CIE illuminants are LED-V1 for A and LED-V2 for D50 and D65, with 1.23, 1.07, and 1.57 units, respectively. The work studied is a Moorish epigraphic frieze of plasterwork with a tiled skirting from the Nasrid period (12th–15th centuries) exhibited in the Museum of the Alhambra (Granada, Spain).

## 1. Introduction

Spectral imaging has become a state-of-the-art tool in many fields of research [1]. New portable and fast imaging devices, together with powerful computers, enable researchers to retrieve valuable information from spectral images. Within the field of spectral imaging, applications related to art and cultural heritage are increasingly taking advantage of this promising technology [2,3,4].

With spectral information, scientists can develop many different applications where color can be handled without constraints regarding the selection of a fixed illuminant or a given spectral sensitivity of the imaging system. In this regard, new solid-state lighting products are rapidly filling the lighting market. In particular, white light-emitting diode (LED) sources are replacing banned incandescent lamps and other lighting technologies in most general lighting applications because of their high efficiency at a low cost. However, the spectral power distributions (SPDs) of white LEDs differ markedly from those of conventional light sources. This fact raises questions concerning possible modifications of color appearance of specific objects such as human skin. Such matters can be addressed by comparing color rendition properties of modern white LEDs with those of lamp types that LEDs are intended to replace [5,6].

LEDs are nowadays the first choice when dealing with art-related applications because their spectral characteristics allow the visible illumination of art and heritage works without radiating them with harmful UV or IR light. This was studied by Luo et al. [7] for the specific case of photographic material displayed in museums, though the same problems can be found for artworks such as paintings or ceramics. Several authors have studied LED illumination for museums in terms of its color appearance. However, as pointed out in a study by Garside et al. [8], although illumination is a key factor in museum management, there is a lack of either standardization or sharing of best practices. This leads to a great variability in the illumination selection process.

In a study by Berns [9], the possibility of using white LED illumination composed of RGB (Red, Green, Blue) LED combinations for museum lighting was analyzed, adapting some chosen primaries to match D65 chromaticity over 24 reflectance samples. Soltic and Chalmers [10] used 14 color samples and mixed LED spectra to create white LED illumination that minimized CIEDE2000 color differences compared to CIE (Commission Internationale de l’Éclairage) standard illuminants A and D65, and CIE illuminant D50. Amano, Linhares, and Nascimento [11] made use of spectral imaging using a liquid crystal tunable filter (LCTF) attached to a monochrome camera, to study the color constancy of postcards reproducing real works of art under different illuminants (including LEDs). They studied the spectral and color differences, as well as color constancy in pseudo-randomly selected local areas of skin and non-skin regions.

Several works have also reported the results of psychophysical experiments investigating how illuminance levels and correlated color temperatures (CCT) of the illumination affect observer preferences, especially for museum lighting [12,13,14,15].

The present study used the hyperspectral image capturing and processing techniques presented by Martínez et al. [2], but rather than colorimetrically studying a particular piece of art, we aimed to use hyperspectral imaging technology in order to present a complete approach for the pixelwise quantitative and graphic analysis of the final color appearance of art or heritage works displayed under a selected illuminant. We considered for this study only one nearly flat art piece, though the whole workflow presented here can be used for similar pieces. In addition, as far as our knowledge goes, this is the first study available in which CIE LED illuminants [16] are studied and compared with classical CIE illuminants such as A, D50, and D65, within the context of cultural heritage. The methods and results presented in this manuscript are not intended for setting a standard in museum lighting. Nevertheless, we feel they can be useful to professionals working in art and cultural-heritage science [8], who usually use point-measurement-based devices such as spectrophotometers [17]. The broad potential of these results opens the possibility of a more thorough study of the colorimetric characteristics of pieces of art under arbitrary illuminants.

## 2. Materials and Methods

### 2.1. The Art Piece

The work studied consists of a portion of a Moorish epigraphic frieze of plasterwork and a tile skirting panel from the Nasrid period (Nasrid Kingdom of Granada, 1238–1492) and later Moorish additions (14th–16th centuries). It is on permanent display in the Museum of the Alhambra (Palace of Carlos V of the Alhambra, Granada, Spain), with inventory number R-1612. From a larger wall section measuring 152.5 cm high and 128.5 cm wide, the portion analyzed in this study measures 40 cm × 60 cm. Its original location was presumably the southern hall of Comares Palace (Hall of the Myrtles) [18,19]. Thus, it was most likely that its original illumination was mainly indoor illumination. This artwork was chosen for being representative of both plasterwork and tiling from the Islamic tradition.

**Figure 1:**
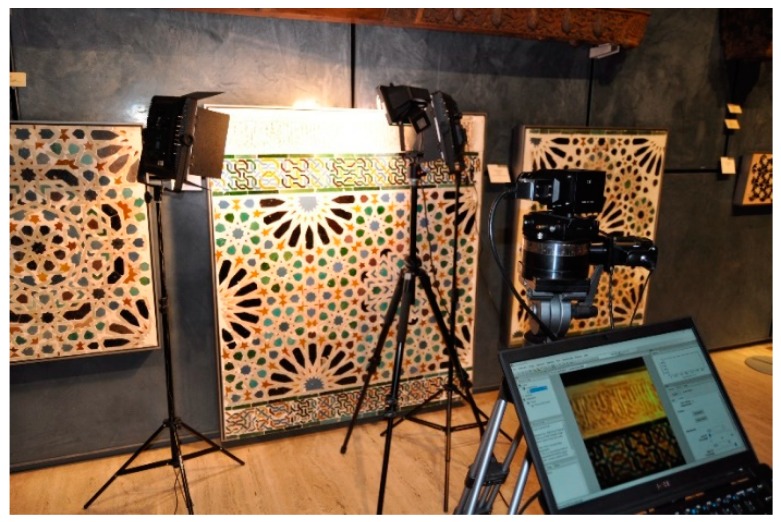
Full piece studied shown in situ with the illumination and image-capturing system.

The plaster frieze at the top of the panel bears epigraphic decoration in Nasrid calligraphy and is framed in the upper and lower parts by a double interlocking motif [20]. Below the frieze is a polychrome tile skirting made by the tiling technique called zellige, which consists of glazed tiles composed of pieces (tesserae) of varying complexity [21]. In this case, the decorative geometric design is formed by thick black, blue, green, and yellow curvilinear lines interlocking to form repeating designs over a white background. Figure 1 shows the complete piece, as well as the illumination and capturing system used in this study (described in Section 2.3.1.).

Although no specific materials analysis on the plaster frieze are available at the moment, it is possible to make an approximation of its composition taking as reference the analysis of other or similar plasterwork. The samples analyzed in a plasterwork from the Hall of the Mexuar (Mexuar Palace, Alhambra de Granada), examined by polarized light microscopy (PLM) and scanning electron microscopy-energy dispersive X-ray (SEM-EDX) show two white layers divided by an intermediate reddish-orange layer. The white layers are made of gypsum (hydrated calcium sulphate, –CaSO_4_. 2H_2_O–) as the main component, in addition to calcium carbonate (CaCO_3_), clays, and iron oxides. In the reddish-orange layer, silicon, aluminum, potassium, iron, calcium, and sulfur were identified, which suggests the presence of iron clays, gypsum, and calcite [22]. 

In the same way, no chemical analyses on the panel of tiles are available. However, we made colorimetric and chemical analyses on tilework from the Courtyard of the Maidens of the Real Alcázar of Seville [23,24,25], whose tiles were also made by Nasrid crafters, much like the Alhambra ones. The referred analyses by scanning electron microscopy-energy dispersive X-ray (SEM-EDX) confirm the presence of lead-based glazes with different metal elements that make oxides. These are the main chemical elements identified that add color to the glazes: copper (green), manganese (black), tin (white), cobalt (blue), and iron (yellow-orange). Silicon always appears as a vitrifier and lead as a flux.

### 2.2. Illuminants

The nine LED illuminants recently recommended by CIE [16] represent current typical white LED lamp spectra. They were determined from experimental measurements of spectral power distributions of 1298 white LED sources available in the market [26]. Table 1 shows the main colorimetric data of the nine CIE LED illuminants: CIE *x*, *y* chromaticity coordinates, correlated color temperature (CCT), distance to the Planckian locus in the *u*, *v* diagram (∆*uv*), CIE general color rendering index (*R*_a_) [27], and CIE 2017 color fidelity index (*R*_f_) [28]. The positive/negative signs in ∆*uv* indicate that the illuminant is positioned above/below the Planckian locus in the *u*, *v* diagram. The values of *R*_a_ and *R*_f_ are in the range of 0–100. The CIE illuminants B1 to B5 represent typical phosphor-converted blue LEDs at different CCTs, which are the most commonly used. The CIE illuminant BH1 is representative for white LEDs that consist of a mixing of phosphor-converted blue LED and red LED (also called a blue-hybrid LED). The CIE illuminant RGB1 represents typical spectral shapes for mixing of red, green, and blue LEDs. Finally, the CIE illuminants V1 and V2 represent typical spectral shapes for phosphor-converted violet LEDs at two different CCTs. Currently, LED white sources similar to RGB1, V1, and V2 are not commonly used [26].

**Figure 2:**
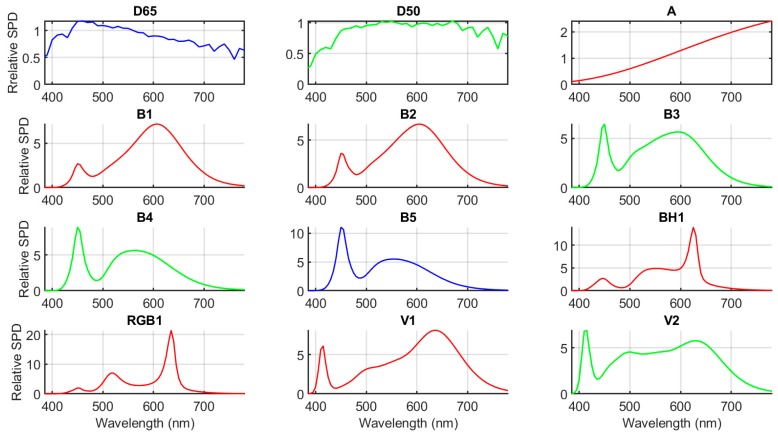
Normalized spectral power distributions (SPDs) of the 12 CIE illuminants studied (*Y* = 100 for all illuminants).

In addition to the above-mentioned CIE LED illuminants, three classical CIE illuminants were also studied. Specifically, the nine CIE LED illuminants were compared against the CIE standard illuminants A and D65, which are considered representative of indoor and outdoor lighting, respectively. In addition, the CIE illuminant D50 was also considered here because currently CIE Technical Committee 2-82 is preparing a revision of CIE S 014-2, which will include CIE illuminant D50 as a new CIE standard illuminant [16]. The approximate CCTs of CIE illuminants A, D65, and D50 are 2856 K, 6500 K, and 5000 K, respectively. The normalized spectral power distributions (SPDs) of the 12 illuminants studied are plotted in Figure 2. The normalization applied is such that the tristimulus value *Y* equals 100 for each illuminant.

### 2.3. Workflow

**Figure 3:**
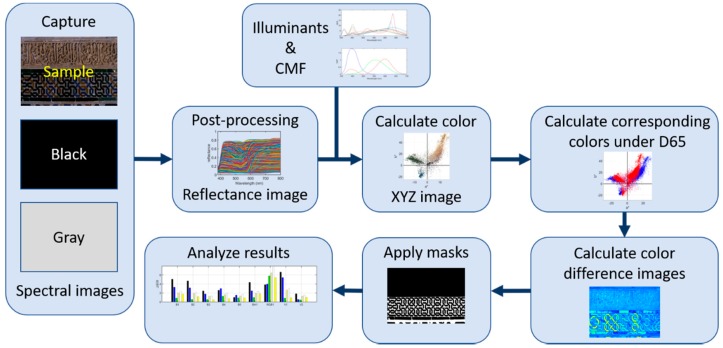
Workflow scheme from the spectral image capture for the analysis of final color-difference results. The small figures in each step are just meant for illustrating them.

This section is divided into five subsections, each explaining one stage of the overall capturing and processing workflow. A scheme of this workflow is shown in Figure 3.

#### 2.3.1. Spectral Image Capture and Post-Processing

The spectral images represented below were captured using a hyperspectral image scanner model Resonon Pika L. The linear sensor size was 900 pixels. The operating wavelength range was from 380 to 1080 nm. The spectral resolution after applying hardware binning was 4.1 nm. Since this study focuses on color differences, only the range from 380 to 780 nm was used.

The light sources used to illuminate the sample were two 36 W Cromalite Nanguan CN-600CSA LED (Nanguang Photo&Video Systems Co., Ltd, Shantou, China) panels, which were placed on sturdy tripods at the left and right sides of the sample, and a 500 W halogen lamp. The exposure time used was 28 ms, and the ISO gain was set to 1. This means that each scanned line was exposed for 28 ms. Since the final image was 1160 lines wide, the total capturing time was roughly 32 s. This was enough for the illuminance impinging on the piece, which was 720 lux at the center of the piece.

**Figure 4:**
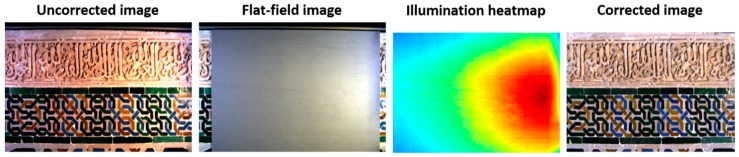
From left to right: sRGB renderings of the original sample, flat uniform gray sample, illumination heatmap over the flat gray sample, and final corrected image under a spatially uniform illumination with CIE standard illuminant D65.

To correct the sensor spectral responses of the imaging system and the spectrally and spatially non-uniform illumination (i.e. the so-called “flat-field correction” explained in [29] and used in [2]), we captured two extra images using exactly the same settings used to capture the image of the sample. These extra images are called the black and gray images. Figure 4 shows the sRGB rendering of the original spectral images captured for the sample and the flat-field uniform gray sample. Also, Figure 4 depicts the heatmap of the illumination over the flat-field sample, and the sRGB rendering of the final flat-filed-corrected image. Note that the corrected image shows only those regions of the uncorrected image covered by the flat-field sample. Hence, the studied corrected image was completely flat-field corrected. It is evident from an examination of the illumination heatmap (where red/blue colors indicate areas with the highest/lowest illuminances), that the illumination over the sample is not spatially uniform (nor is it spectrally uniform). Hence, flat-field correction becomes necessary to gain a realistic and useful spectral reflectance image.

#### 2.3.2. Color Calculation

**Figure 5:**
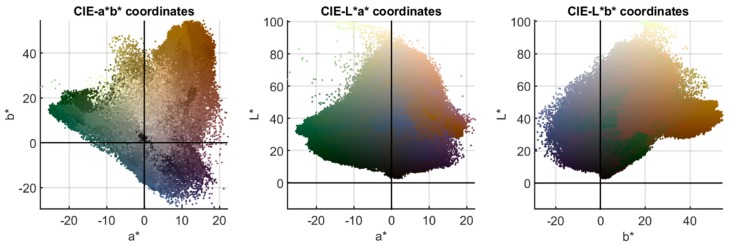
L*, a*, b* distributions for the corrected image in Figure 4 (CIE standard illuminant D65).

After computing the spectral reflectance image (last plot in Figure 3), we calculated the *XYZ* tristimulus values for each pixel using the CIE 1931 standard observer and the 12 different illuminants explained in Section 2.2. From *XYZ* tristimulus values, the transformations to different color spaces are straightforward. CIE L*, a*, b* images were calculated, as were sRGB images. As an example of the results found, Figure 5 shows the three two-dimensional projections in the L*, a*, b* color space of the color image under the CIE standard illuminant D65, using true color for the dots. Sometimes researchers compare these kinds of plots for two different illuminants, seeking information on the perceptual changes in the appearance of a given sample under two illuminants. However, this practice requires the origin of coordinates for any of these plots under two different illuminants to be the same, and therefore we must use the technique of “corresponding colors” described in the following section to plot in CIELAB the results for two different illuminants. 

#### 2.3.3. Corresponding Color Calculation

“Corresponding colors” are defined as pairs of color stimuli (e.g., two sets of different tristimulus values) that have the same color appearance when one is seen under one set of adaptation conditions (e.g., a first illuminant) and the other is seen in a different set (e.g., a second illuminant) [30]. Both stimuli come from the same object color under two different illuminants. This is not the same as illuminant metamerism, which studies how the color between a pair of different object colors change when the illuminant is changed. It is used as a reference illuminant with the same CCT as the source to compute the CIE general color rendering index [27] or the CIE color fidelity index [28] of a light source. However, in this article we are studying color inconstancy when changing from a CIE LED illuminant to one of the three main CIE illuminants (A, D50, and D65). This is done by calculating corresponding colors before using the CIEDE2000 color-difference formula. In the current paper, the computations of corresponding colors were made from all studied illuminants to D65, using the CAT16 (Chromatic Adaptation Transform) model [31], assuming an average surround, complete adaptation (*D* = 1), and adapting luminance *L*_A_ = 64 cd/m^2^ (typical value for surface color evaluation in a light booth). For the purposes of the current study, the results found using the CAT16 model can be considered equivalent to those using the current CIE-recommended model, CAT02 [32]. Nonetheless, the comparison between the accuracies of both CATs is out of the scope of this article. Hence, in this study, the most recent CAT16 model was considered.

#### 2.3.4. Color-Difference Calculations

When the same object color is studied under two different illuminants, a color-difference formula like CIELAB or CIEDE2000 cannot be used since there are two different reference whites, one for each illuminant. After the calculation of the corresponding colors under D65, there is only one reference white point and hence the CIEDE2000 color differences (Δ*E*_00_) [33] can be computed. We calculated them pixelwise in order to compare how much the final color of the sample changed when using the nine CIE LED illuminants compared to the classical CIE illuminants (A, D50, and D65). Then, each LED illuminant was compared against A, D50, and D65. This color-difference formula was chosen because it is the latest recommendation made by CIE and ISO as a standard [34]. In fact, CIEDE2000 was initially recommended for a set of “reference conditions” (e.g., uniform samples with CIELAB color differences below 5.0 units) which are different from the current ones. However, different authors have employed the CIEDE2000 color-difference formula under many different viewing conditions, including complex images as test samples [35,36,37].

#### 2.3.5. Masking

**Figure 6:**
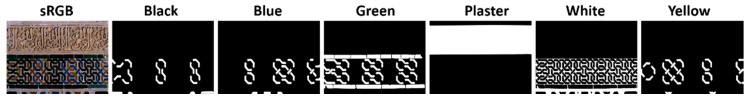
Binary masks used to determine the CIEDE2000 color differences from different color regions.

To perform a more detailed analysis of the sample, we also studied the CIEDE2000 color differences separately by colors. The sample studied in this work has six main colors, denoted as black, blue, green, plaster, white, and yellow. To separate them, we manually segmented binary masks, as indicated in Figure 6, in order to extract the color difference information only from the desired color in each case (white areas), disregarding the rest (black areas). The top plaster region was an area (plaster). For the ceramic tiles’ region, despite some variability among different tiles, in the current piece it was easy to make the segmentation into the five different colors by visual inspection (mainly hue inspection).

## 3. Results

This section is divided into two subsections. First, the sRGB renderings are plotted for visual comparisons of color appearance under the nine CIE LED illuminants and the three main CIE illuminants (A, D50, and D65), considering their corresponding CCTs. Then, the CIEDE2000 color differences are presented in two different formats for each pair of illuminants and for each color of the sample.

### 3.1. Visual Comparisons of Color Appearance and Related CCTs

**Figure 7:**
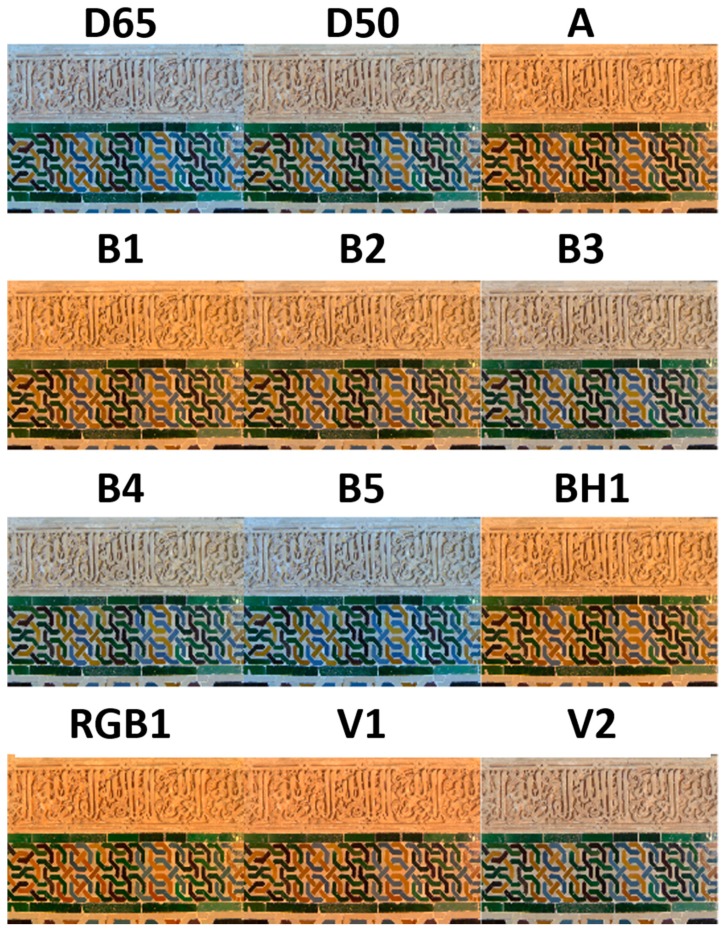
The sRGB rendering of the sample under the 12 CIE illuminants studied.

Figure 7 shows the sRGB images rendered using the 12 different illuminants. This simulation is a powerful tool for art and heritage professionals when deciding the illumination to be used over a specific piece in a museum. In this way, the final color appearance of the sample under the different illuminants can be viewed before the illuminant is finally chosen.

At a glance, the reddish appearance of the piece can be appreciated under five of the LED illuminants: B1, B2, BH1, RGB1, and V1. These illuminants have CCTs below 3000 K. Hence, they look closer to the appearance under CIE standard illuminant A. Then, the LED illuminants B3, B4, and V2, which have CCTs ranging from roughly 4000 K to 5000 K, resemble the CIE illuminant D50. Furthermore, the LED illuminant B5, with a CCT of 6598 K, takes on a color appearance similar to that of CIE standard illuminant D65. In any case, as will be shown below, for images under two different illuminants, the minimum difference in their CCTs is not always equivalent to the minimum average color difference in CIEDE2000 units. Specifically, for the main CIE illuminants A, D50, and D65, the CIE LED illuminants with the lowest average CIEDE2000 color differences are V1 and V2 (see Section 3.2), while the ones with the lowest CCT differences are BH1, B4, and B5, respectively.

**Figure 8:**
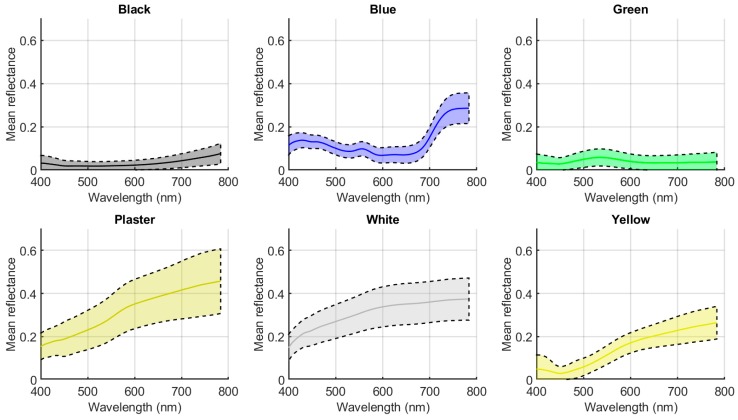
Mean spectral reflectances (and standard deviation) of the six color regions studied (manually segmented using masks shown in Figure 6).

In addition, a close examination of the plots shown in Figure 8 reveal several differences for images under illuminants with very similar CCTs. This is not surprising because CCT is only a rough approach to the color of a white source (e.g., cool/warm source), while the specific spectral power distributions of light sources are the essential factors in the determination of the overall appearance of an image.

### 3.2. Color-Difference Analysis

Figure 8 plots the mean spectral reflectances (and their standard deviation) measured for the six different colors studied. Using the masks shown in Figure 6, all reflectances under the white pixels of the mask were averaged for each color. As expected from visual inspection of the images in Figure 7, plaster was the region with highest standard deviation since the heterogeneity in this not completely flat region is high.

**Figure 9:**
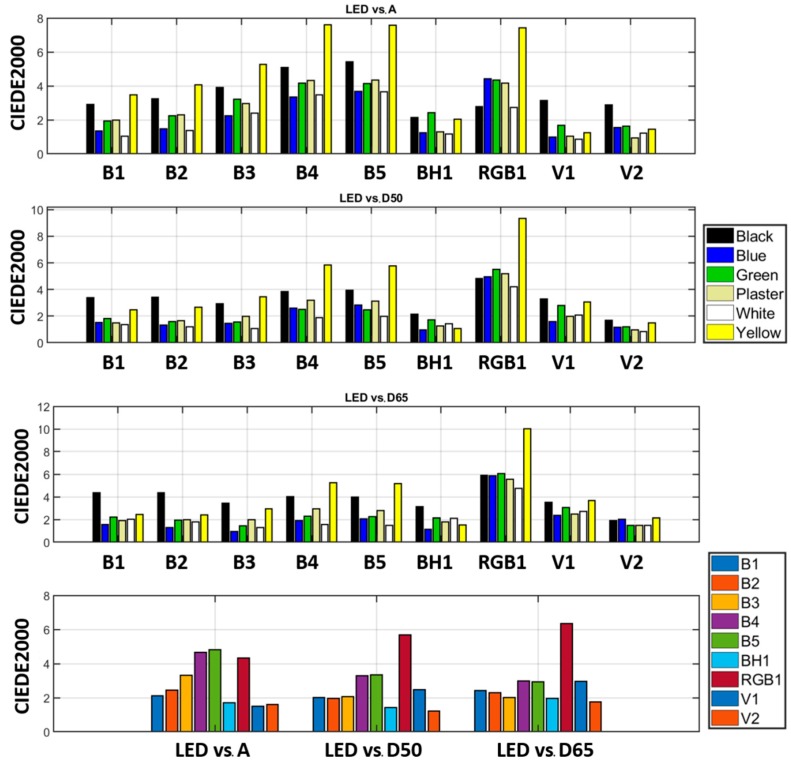
CIEDE2000 color differences for each CIE LED illuminant against CIE illuminants A, D50, and D65 for each color of the sample. The bottom row shows the average color differences for all colors of the sample.

Figure 9 shows bar plots of the CIEDE2000 color differences calculated for each LED illuminant and each color of the sample against illuminants A (top row), D50 (second row), and D65 (third row). The bottom row in Figure 9 shows the average color differences for all the colors of the sample.

Figure 9 shows that different CIE LED illuminants would be selected depending on which of the CIE illuminants A, D50, and D65 we wish to match. For the sample studied, using the criterion of the minimum average CIEDE2000 color difference, we found that the best CIE LED illuminant to match standard illuminant A was V1, since it presents the lowest average color difference (1.23 CIEDE2000 units). On the contrary, for matching D50 or D65, we chose V2 in both cases (1.06 and 1.57 CIEDE2000 units, respectively).

As a reference, in previous studies the visual threshold value to detect average color differences in images was about 2.0 CIELAB units [38], while the acceptability value for complex images may be around twice that [39]. While there is no scale factor to transform from CIELAB into CIEDE2000 units, for uniform samples, 1.0 CIELAB unit is equivalent to approximately 0.6 of a CIEDE2000 unit [40].

Nonetheless, analyzing the color differences color-wise, we can appreciate differences concerning the most suitable CIE LED illuminant to choose depending on the color of the sample. For instance, in an effort to mimic the D65 illuminant (third row), V2 is the CIE LED with the lowest average CIEDE2000 color difference for the whole sample (1.57 CIEDE2000 units) and for individual colors it yields the best results only for black and plaster (1.92 and 1.47 CIEDE2000 units, respectively). However, for blue, green, and white, B3 performs best (with 0.94, 1.45, and 1.28 CIEDE2000 units, respectively), whereas for yellow, BH1 would be the best choice (with 1.52 CIEDE2000 units).

**Figure 10:**
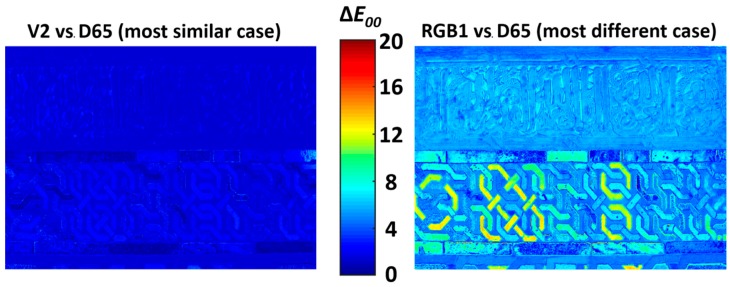
CIEDE2000 color-difference heatmaps showing the magnitude of differences between D65 and V2 (**left**) and between D65 and RGB1 (**right**), which correspond to the best and worst average D65 matches for our sample, respectively.

For a more complex piece where the different color areas are not so clearly segmented, this information is easier to visualize using a color-difference heatmap which indicates the areas of the image that present higher or lower color differences in comparisons of two illuminants. This is the case shown in Figure 10, which presents the color-difference heatmaps comparing the illuminant D65 with the best average (V2, left) and worst average (RGB1, right) LED illuminants. Both heatmaps are equally scaled in order to show the differences between them, using the scale shown in the center of Figure 10. We see that the heatmap on the left represents weaker color differences (which is darker and bluer) than the heatmap on the right (which is lighter and yellower). Note that the comparison in this case is made pixelwise between the image under the illuminant D65 and the image under the aforementioned CIE LED illuminants.

A closer examination of the right heatmap indicates that areas with the highest color differences (CIEDE2000 of approx. 10 units, represented with yellowish colors), correspond to the yellow areas of the sample, which are the ones with the strongest color difference in Figure 9 (third row, illuminant RGB1).

This methodology provides a powerful tool to visualize the regions of a specific sample, where a given illuminant proves better or worse at matching the color appearance of another illuminant.

## 4. Conclusions

In this work, we have shown how spectral imaging with a hyperspectral imaging scanner can be used to represent a spectral reflectance image of an artwork and simulate its colorimetric attributes pixelwise. This technology enables an exhaustive analysis of the color appearance under a selected illuminant. The complete workflow is presented and explained, and results are shown with a real historical work from the Museum of the Alhambra (Granada, Spain).

The sample was studied under nine CIE LED illuminants and compared with the classical CIE illuminants, A, D50, and D65. These comparisons were made possible using the calculations of the corresponding colors with a chromatic adaptation transform analogous to CAT02. The results reveal that, for the studied sample, in terms of minimum average CIEDE2000 color differences, V1 illuminant is the best choice for matching the A illuminant, and V2 is the best choice for matching both the D50 and D65 illuminants.

However, even though the CCT of each illuminant was demonstrated to give some information of the overall appearance of the sample under it in terms of warmer or cooler color appearance, the CIE LED illuminants with CCTs closest to the main CIE illuminants were not the same with the closest average CIEDE2000 color differences.

We have also shown how, for the sample studied, even if the above-mentioned CIE LED illuminants were on average the best choices for matching the A, D50, and D65 illuminants, when the piece was studied locally using a masking technique, different colors of the sample presented different CIE LED choices with lowest CIEDE2000 color differences. This information was easily discerned by means of color-difference heatmaps that show these differences pixelwise.

## Figures and Tables

**Table 1:** The main colorimetric data of nine light-emitting diode (LED) illuminants recommended by the CIE [16].

CIE LED	B1	B2	B3	B4	B5	BH1	RGB1	V1	V2
*x*	0.4560	0.4357	0.3756	0.3422	0.3118	0.4474	0.4557	0.4548	0.3781
*y*	0.4078	0.4012	0.3723	0.3502	0.3236	0.4066	0.4211	0.4044	0.3775
CCT (K)	2733	2998	4103	5109	6598	2851	2840	2724	4070
∆*uv* (× 10^3^)	−0.7	−1.0	−0.7	+0.5	+0.9	−0.3	+4.3	−1.9	+1.0
*R*_a_	82	83	85	77	80	92	57	95	96
*R*_f_	84	84	85	77	79	85	71	87	94
